# Supplementary material for: Association between body mass index and atopic dermatitis among adolescents: Findings from a national cross-sectional study in Korea
Source: PLoS One. 2024 Jul 19;19(7):e0307140. doi: 10.1371/journal.pone.0307140 (PMC11259265; doi:10.1371/journal.pone.0307140)
Supplement: Attachment — Submitted filename: Response to reviewers.docx [file pone.0307140.s001.docx]

**Revision note for the manuscript ID: PONE-D-24-07867**

**Association between degree of obesity and atopic dermatitis among adolescents: Findings from a national cross-sectional study in Korea**

We express our gratitude for the opportunity to make revisions to our manuscript. Throughout the revision process, we have taken into careful consideration the feedback provided by the editor and reviewers, and have made every effort to integrate their suggestions appropriately. Following the given instructions, we have endeavored to elucidate the modifications implemented in response to all reviewer comments. The insights offered by the reviewers were beneficial, and we value the constructive criticism provided for our submission. Upon addressing the raised concerns, we believe that the quality of the paper has significantly improved, and we trust that you share this sentiment. Our detailed responses to each comment are outlined below, and we have enclosed a revised version of the manuscript with the tracked changes. Once again, we appreciate the insightful and constructive feedback.

**Response to Editorial Board’s comments**

Thank you for submitting your manuscript. The reviewers and I believe it is of potential value for our readers. However, the reviewers have raised a number of very important issues, and their excellent comments will need to be adequately addressed in a revision before the acceptability of your manuscript for publication in the Journal can be determined. We cannot guarantee that your revised paper will be chosen for publication; this would be solely based on how satisfactorily you have addressed the reviewer comments.

1. KCDC → KDCA

2. To determine the association between BMI and AD, multiple logistic regression analysis was performed after adjusting for all covariates. → Please cite the statistical guideline (DOI: <https://doi.org/10.54724/lc.2022.e3>).

- *We offer our sincere apologies for any confusion. The pertinent text has been revised to adhere to the data usage guidelines, and we have included references to statistical articles relevant to the analysis conducted. Your meticulous review is greatly valued.*

**Revised manuscript with track changes, page 3, 22**: KCDC → KDCA, The Korean Disease Control and Prevention Agency

**Revised manuscript with track changes, page 4, 5**: KCDC → KDCA

**Revised manuscript with track changes, page 6**: To determine the association between BMI and AD, multiple logistic regression analysis was performed after adjusting for all covariates [28].

- **Added reference**: Lee SW. Regression analysis for continuous independent variables in medical research: statistical standard and guideline of Life Cycle Committee. Life cycle. 2022;2.

**Response to Reviewer #1’s comments**

**Point 1**

The title might be revise as "Association between BMI and atopic dermatitis among adolescents: Findings from a national cross-sectional study in Korea".

- *Thank you for your good comment. In this study, the comparison between underweight and normal weight is a crucial aspect to consider. Therefore, it is imperative to have a title that thoroughly encompasses all weight categories, including this comparison. In this regard, we wholeheartedly concur with your perspective and are confident that the updated wording of the title will better articulate the primary focus of our study.*

**Revised manuscript with track changes, page 1**: Association between body mass index and atopic dermatitis among adolescents: Findings from a national cross-sectional study in Korea

**Point 2**

Background part: I suggest describe the epidemical situation of atopic dermatitis at first.

- *Thank you very much for your insightful comment. The flow of describing the epidemiological situation looks more natural after the definition of AD. We rearranged the order inside the first paragraph of the introduction.*

**Revised manuscript with track changes, page 3**: Atopic dermatitis (AD), also known as atopic eczema, is a common chronic inflammatory skin disease characterized by relapsing eczematous lesions and intense itching. Internationally, the incidence rates of AD are approximately 15%–20% in children and 1%–3% in adults. The Korean Disease Control and Prevention Agency (KDCA) conducted a survey on the prevalence of AD in adolescents, revealing a rate of 16.4% among individuals aged 12-18 years.

**Point 3**

Methods: part: Please describe the measure methods of the important variables. How to measure obesity and underweight?

- *Thank you for highlighting these crucial points. We apologize for not providing sufficient information on how to measure BMI. We have noted in the variable description paragraph that BMI is measured using the responses of adolescents who participated in the survey.*

**Revised manuscript with track changes, page 5**: These were obtained by the response that adolescents who participated in the survey recorded their height and weight to the first decimal place.

**Point 4**

Please add the inclusion criteria and exclusion criteria in the part of Participants.

- *Thanks you very much for your meaningful comments. We have clarified the final inclusion criteria in the “Participants” section by outlining the exclusion criteria applied to the initially qualified participants. Through this approach, we believe that readers can gain a clearer comprehension of the participants included in the study.*

**Revised manuscript with track changes, page 5**: From the 3-year data, all 167,099 participants were initially considered eligible. After excluding adolescents who were missing in height and weight (N=4,372) and adolescents who were missing in other variables (N=18,589), a total of 144,138 participants were finally included in this study.

**Point 5**

Results part: Please add to the subtitle of different part.

- *Thank you for your valuable comments. We have added subheadings for distinct analysis results in the Results section. Your thorough feedback made it easier for readers to distinguish between the different analyses.*

**Revised manuscript with track changes, page 8**: Descriptive analyses

**Revised manuscript with track changes, page 11**: Regression analyses

**Revised manuscript with track changes, page 13**: Subgroup analyses

**Point 6**

Discussion part: please add to advantage of the research before the limitation of the research.

- *We sincerely appreciate your remarks. Based on your comments, we have emphasized the study's importance by highlighting its strengths before its limitations. Additionally, we have addressed the mentioned overlaps in the conclusion paragraph. Thank you once again for reviewing our work and providing valuable feedback.*

**Revised manuscript with track changes, page 20**: This study has the advantage of being able to generalize to Korean adolescents using nationally representative KYRBS data. In addition, it is significant in that several analyses were conducted to interpret the association with underweight, which was rarely noted in previous studies.

**Response to Reviewer #2’s comments**

**Point 1**

In the Materials and Methods section, under the subsection "Data," the authors mentioned "sampling design method." I intend to question the appropriateness of this sampling technique.

- *Thank you for your valuable comments. We agree that the original sampling description for KYRBS provided in the “Data” section is insufficient. The sampling design method of KYRBS is as follows:*

*“*Multistage cluster sampling design is used by KYRBS to guarantee South Korean sample representativeness. The population was stratified into 117 strata using 39 regional groupings and school levels as stratification factors in order to minimize sampling error. In 17 provinces, regional groups divided into metropolitan, medium or small cities, and rural areas, and then into 39 categories based on factors like living conditions, smoking and drinking rates, population density, and accessibility to schools. During the sample allocation step, the sample size was set to 400 middle schools and 400 high schools. The proportionate distribution approach was used to match the population composition ratio with the sample composition ratio for each stratification variable, and the number of sample schools was dispersed based on provinces, city sizes, and school type. For sampling, the stratified clustered extraction approach was utilized, with school as the first extraction unit and class as the second*.”*

*This is outlined in the KYRBS guidelines, and we hope the explanation of the sample design method used in this data has been sufficient. Additionally, we have included extra information about sampling method in the “Data” section to address any similar questions that readers might have.*

**Revised manuscript with track changes, page 4:** The multistage stratified random cluster sampling technique was employed by the KYRBS to produce a sample that was nationally representative. To reduce sampling error, the sample was stratified by school type and geographic location (17 provinces). 400 middle schools and 400 high schools were selected annually for the sample allocation using proportional sampling in order to match the population.

**Point 2**

Under the subheading "Variables," the study mentioned using BMI levels categorized as underweight, normal weight, and obesity. However, the World Health Organization classifies BMI into underweight, normal, overweight, and obese categories. Therefore, it would enhance the study's credibility to align with the globally accepted classification system.

- *We greatly appreciate your excellent suggestion. We fully agree with your insightful comment that categorizing according to international standards can enhance the study's credibility. Consequently, we have changed the term ‘obesity’ to ‘overweight’ throughout the study, including in the tables and figures, except where it appears in its original context.*

**Revised manuscript with track changes, page 2, 6, 8, 11, 13, 14, 16**: **Overweight**

**Revised Figure: (figure legend) Overweight**

**Point 3**

Atopic dermatitis is a chronic skin condition. However, in this study, participants with a past history of atopic dermatitis (more than 1 year ago) were categorized into the AD "no" group. I believe this could potentially underestimate the prevalence of the disease, which might impact the subsequent analyses conducted in the study.

- *We sincerely appreciate your critical and insightful comment, as the topic you raised is of utmost importance. We have defined the incidence of AD as adolescents with a doctor's diagnosis within a year, recognizing the uncertainty surrounding the prevalence of adolescents diagnosed more than a year ago. Additionally, considering the fluctuations in BMI during adolescence, it's possible that the status of BMI may have changed since their previous diagnosis of AD. Therefore, including adolescents diagnosed in the past but not recently in the analysis may yield inadequate results due to mixed current prevalence. Figure 1 addresses some of these concerns, revealing no significant association among male adolescents previously diagnosed with AD. We believe this supports the previously indicated reasoning. Furthermore, separate results for current and prior diagnoses in Figure 1 enhance the robustness of the main finding by addressing uncertainties regarding present prevalence. We hope this explanation clarifies the matter sufficiently.*

**Point 4**

In the majority of cases, atopic dermatitis (AD) has an onset before the age of five years. However, this study targeted participants aged 12 to 18 years. This age group, coupled with the exclusion of participants with a history of AD, could have influenced the results, particularly in terms of prevalence.

- *We appreciate your thoughtful comment, which aligns with the previous crucial remark you shared with us. Given that the participants in KYRBS ranged in age from 12 to 18, it is unfortunate that other age groups, particularly those under the age of five, the primary epidemic period, could not be included. Similar to previous responses, there is concern that including individuals previously diagnosed with AD but not recently undiagnosed may lead to an overestimation of current AD prevalence. Nevertheless, the sensitivity of the main results was supported by the relevant subgroup analysis. We also believe it is meaningful to include information on effective interventions to investigate persistent influencing factors beyond the primary age of occurrence. Once again, we sincerely thank you for prompting us to consider these aspects.*

**Point 5**

The authors stated that "All participants signed an informed consent when they participated." However, it's worth noting that the majority of participants are minors. This raises ethical concerns regarding whether it is appropriate for a study to involve minors signing consent forms.

- *We are grateful for your thorough review and insightful comment. Research involving minors often requires heightened ethical sensitivity. While it's crucial to note that prior to the survey, data collection obtained approval from both a parent or legal guardian and the adolescent's own consent, it seems that the wording may have been misconstrued, implying only the adolescent's consent was obtained. We have revised the text to prevent any misunderstandings. We appreciate your emphasis on the crucial aspect.*

**Revised manuscript with track changes, page 5**: When participating in the KYRBS, all participants, as well as their parents or legal guardians, completed an informed consent.

**Point 6**

The data provided in the manuscript are publicly available on the website mentioned. However, it's important to note that all the data are in a language other than English. If feasible, it would be beneficial to have the data translated into English for wider accessibility and comprehension.

- *We greatly appreciate your valuable comment. As you highlighted, providing information in English can enhance user accessibility. Regrettably, upon checking, we found no option button available to set the language to English on the relevant web page. Currently, it seems that the only option is to utilize the integrated translation mechanism within the accessing apps. However, if feasible, we will reach out to the department responsible for the website to inquire about the possibility of offering an English version.*
